# Analysing frequent extreme flood incidences in Brahmaputra basin, South Asia

**DOI:** 10.1371/journal.pone.0273384

**Published:** 2022-08-22

**Authors:** Amit Kumar, Subhasree Mondal, Preet Lal

**Affiliations:** 1 Department of Geoinformatics, Central University of Jharkhand, Ranchi, Jharkhand, India; 2 Department of Civil and Environmental Engineering, Michigan State University, East Lansing, MI, United States of America; Đại Học Duy Tân: Dai Hoc Duy Tan, VIET NAM

## Abstract

The present study is focused on the flood inundation in Brahmaputra Basin, which is one of the most recurrent and destructive natural disasters of the region. The flood inundation was assessed using C-Band Sentinel 1A synthetic aperture radar (SAR) during 2015–2020 with precipitation patterns, runoff discharge, and their impacts on land cover in the basin. The study exhibited a very high precipitation during monsoon in the upper catchment resulting in severe flood inundation in downslopes of Brahmaputra Basin. A very high (900–2000 mm) to extremely high (>2000 mm) monthly cumulative precipitation in the south and south-eastern parts of basin led to high discharge (16,000 to 18,000 m^3^s^-1^) during July-August months. The river discharge increases with cumulative effects of precipitation and melting of snow cover during late summer and monsoon season, and induced flood inundation in lower parts of basin. This flood has largely affected agricultural land (>77% of total basin), forests (~3%), and settlement (426 to 1758 km^2^) affecting large wildlife and livelihood during 2015–2020. The study highlights the regions affected with recurrent flood and necessitates adopting an integrated, multi-hazard, multi-stakeholder approach with an emphasis on self-reliance of the community for sustenance with local resources and practices.

## 1. Introduction

The trend of global warming induces longer conditions of no rain followed by a sudden bout of excessive precipitation, causing extreme weather events particularly floods [1–3]. The increasing temperature produces more energy in the Earth’s system, specifically increasing the probability of evaporation from the surface water and oceans augmenting cloud formation [4]. At higher temperatures, the air can hold more moisture content that tends to rise in the precipitation intensity, its duration, and frequency [5–7]. Therefore, floods are triggered by intense precipitation, anomalous longer duration, close repetition of precipitations, topographical characteristics or a combination of all these phenomenon [8–11]. For example, an extended period of very heavy precipitation leads to the development of a low-pressure system apart from extended monsoon depression during the Kerala flood in 2019 [12–14].

In the twenty-first century, floods emerged as one of the most frequent and dangerous natural hazards worldwide [15–18]. from the flood mostly occurs due to torrential precipitation, but there are various factors such as poor drainage system and water storage management, rapid land transformation due to anthropogenic influences, river structure and relief of region, which are also major contributing factors augmenting the severity of extreme flood incidents [19–21]. Particularly in India sub-continent flood is a very common natural disaster during the southwest monsoon (June-August) [22]. The combined attribution of monsoonal precipitation and rapid melting of Himalayan glaciers accelerates the downwards flows of water in river during the summer season [23, 24] which makes a very high flow rate of water compared to the capacity of the river [25–27]. Most of the Indian river originating from Himalayan region has similar nature of flow, during late summer and monsoon river flow increases by multiple times with combined melting of snow and heavy precipitation. Among all the different rivers originating from Himalayan regions, Brahmaputra river in eastern India is one of the most affected flood hazard regions in the world [28]. The Brahmaputra basin is affected every year by catastrophic floods. The melting of eastern Himalayan glaciers during and before the Indian summer monsoon intensifies downstream and causes flood in valley of Northeast India [29]. The factors for flood severity apart from high-flow of river includes rampant deforestation [30], high impervious surface expansion are related to anthropogenic influences rising flood severity. Around 4,500 km embankment along the Brahmaputra is enclosed by the rivers that lead to an acceleration in the river flow and often increase the flood susceptibility [31]. The 65% of annual precipitation (being of the order of 165 cm) in the lower basin (Assam) is caused by the South-West monsoon. The major parts of the runoff in the river were introduced through the heavy precipitation (~ 510–640 cm) in Arunachal Pradesh (Abo and Mishmi hill), and moderately high precipitation (250–510 cm) in the Brahmaputra plain. Therefore, in the present study, the spatial patterns of frequent flood inundation in the Brahmaputra basin were analysed for the recent years (2015–2020) and its impacts on the land use/land cover (LULC) were discussed.

## 2. Study area

**Fig 1:**
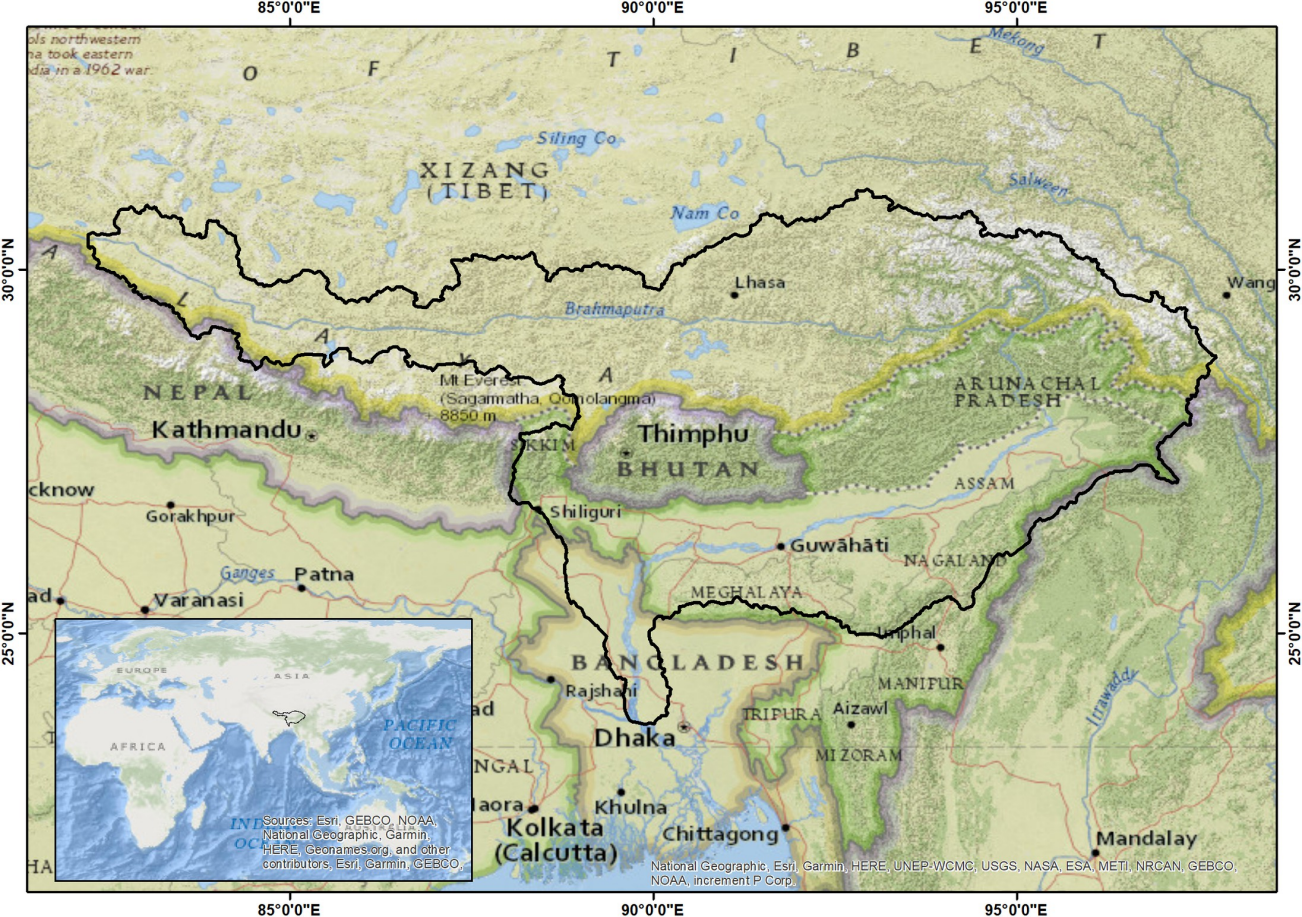
Study area representing Brahmaputra basin for flood inundation assessment (Base map from OpenStreetMap and OpenStreetMap Foundation).

Brahmaputra basin is located in the north-eastern parts of the Indian Subcontinent and lies between 23.9°N to 31.5°N latitude and 82.1°E to 97.7°E longitude (Fig 1). The river Brahmaputra originates from the Angsi glacier located in the south of Tibet (at an elevation of 5300 m) and falls in the Bay of Bengal after travelling 2,880 km through Tibet, India, and Bangladesh. The one-third portion of the Indian Brahmaputra basin is in Assam state of India. The Brahmaputra Valley within Assam consists of vast alluvial floodplains covering an area of about 56480 sq. km (altitude 34130 m). The upper part of the basin lies in the trans-Himalaya (Tibet), greater Himalaya (Nepal and Arunachal Pradesh), middle Himalaya (Arunachal Pradesh), and Shivalik range (lower part of Arunachal Pradesh) as the lower part of Himalaya. The lower and middle part of the basin lies in the low-lying plain valley primarily in Assam (India) and Bangladesh, which formed due to continuous fluvial deposition of Himalayan streams and rivers. There are various subsidiary rivers, which confluence with Brahmaputra River in the low-lying Assam valley including Subansiri (originates in Tibetan region), Kameng (Indo-Tibet border), Beki and Sankosh (northern Bhutan), Manas (southern Bhutan), while rivers Teesta, Rangeet, Rangpo (originates from Sikkim) confluence in Bangladesh. The Brahmaputra and its tributaries in India (Assam), Bangladesh, and Bhutan elucidates intense environmental risk due to climate change and the extensive increase in anthropogenic activities, led to exposure to floods, riverbank erosion, landslide [27].

## 3. Data used and methodology

To deduce the flood inundation and its impact on the Brahmaputra basin, various concurrent SAR imagery for 2015–2020, and ESA-CCI land use/ land cover (300m) were used. The GPM precipitation of 0.1° was used for analysing monthly and the sum of seasonal precipitation (June-September) and anomalous precipitation between 2015–2020, whereas long-term mean (2001–2020) precipitation was used to estimate precipitation anomaly, with reference to the mean of the respective time period from 2000 to 2020. The Global Flood Awareness System (GloFAS) ERA5 river discharge reanalysis products having horizontal grid resolution of 0.1° were used to estimate the river discharge of Brahmaputra in the eastern-lower part of Arunachal and upper part of Bangladesh.

Due to the large basin coverage, the Google Earth Engine (GEE) platform was used to execute the task impeccably for inundation mapping and to deduce flood impact on LULC on a parallel processing architecture with a powerful web platform for cloud-based processing for remote sensing datasets. The Sentinel 1A/1B GRD datasets of the VH polarisation with ascending pass were retrieved from ESA for the concurrent flood during 2015–2020 in the Brahmaputra basin. The orbit correction and noise removal methods were applied to improve the geometric accuracy by ~10 cm [32] and remove border noise and low-intensity noise. Furthermore, radiometric calibration followed by a Lee sigma Speckle filter (5x5 window size) was performed for the qualitative assessment using the backscatter coefficient and making the image more interpretable. The Terrain correction applied to the filtered image employing the Shuttle Radar Topographic Mission (SRTM) one arc-second digital elevation model (DEM) to rectify the distortions including foreshortening, layover, or shadowing effects. The GRD data of Sentinel 1A/1B provided by ESA has geometric distortions due to complex terrain and when the sensor is not pointing directly at the Nadir location so ortho-rectification was necessary before the classification of flood and non-flood data. The ortho-rectification is a substitute of geo-referencing method of remote sensing datasets. It converts images into suitable form for maps by removing the sensor, satellite motion and surface related geometric distortions from Level 1 SAR imagery. Later, pre-processed image backscatter coefficients were converted to decibel. For the Brahmaputra basin, the backscatter intensity of VH varies between -15 to -20 dB during the pre-flood period, while the backscatter intensity was approximately -25 to -30 dB during the post-flood analysis. Backscatter ratio methods were adopted following Lal et al. (2020) [17] and it’s one of the widely used techniques for flood inundation mapping [33, 34]. The backscatter ratio method estimates the changes in due to flood inundation the during flood mosaic backscatter SAR data were divided from pre-flood mosaic SAR backscatter data to deduce the flood inundation. The resultant from backscatter ratio method shows a change between pre-flood and post-flood backscatter coefficient and value of 1.26 selected based on a trial-and-error approach. Using the resultant value binary image was created for flood inundation, value above 1.26 assigned as 1 (flood-inundated pixel) and value below 1.26 assigned as 0 (no-data). The flood inundation binary images show all the permanent water bodies as flood pixels, the JRC Global Surface Water dataset of 30 m resolution was used to refine the flood water classification by masking the surface water bodies. A digital elevation model (WWF Hydro SHEDS, a spatial resolution of 3 arc-seconds) was used to remove blind spots having higher slopes to eliminate its confusion with flooded pixels. Furthermore, the connectivity of the flood pixels is assessed to reduce the noise. ESA-CCI land cover of 300m spatial resolution was used to glean the impact of flood inundation on different LULC classifications during respective years using overlay analysis in the GEE environment.

The code for assessing the flood inundation of any region using Sentinel 1A/1B is available at https://code.earthengine.google.com/ee2cdaeff8adab3f59c8e4a21e6868c1.

## 4. Results and discussion

### 4.1 Land use/ land cover change in Brahmaputra basin

The land use/ land cover change (LULCC) was analysed using the ESA-CCI LULC data set for the Brahmaputra basin covering part of India, Tibet, Bhutan, and Bangladesh during 2000–2019. The study exhibited the dominance of grassland (33.72%; 225905.7 km^2^) primarily in the upper parts of the Brahmaputra basin (Tibet) followed by forest cover (30.19%; 202289.9 km^2^) primarily in the lower Himalayan regions, and agriculture (26.89%; 180160.3 km^2^) primarily in Assam and Bangladesh. The LULC change in the Indian and Bangladesh regions are primarily due to exponential population growth, lack of proper valuation of ecological services, biological limitation, concurrent flood disaster, and improper management of land by the public. The significant decrease was observed primarily in the agriculture (1.58%; 2853.18 km^2^), followed by shrubland (28.66%; 3218.67 km^2^), waterbody (3.76%; 643.41 km^2^), bare land (0.8%; 119.25 km^2^) and sparse vegetation (4.85%; 56.7 km^2^) during 1992–2020. In contrast, increase was evident in settlement (290.16%; 1282.23 km^2^) followed by wetland (4.03%; 25.11 km^2^), forest cover (2.1%; 4240.44 km^2^) and grassland (0.59%; 1343.43 km^2^) (Table 1A). The rise in forest cover and grassland modulates the regional climate as increase in precipitation and decrease in temperature [35].

**Table 1:** (a) Land-use/land-cover change between 2000 and 2019 and, (b) impact of flood on different land-use/land-cover types in Brahmaputra basin.

**(a)**						
**YEAR**	**2000**		**2019**		**Change**	
**LULC **	**Area in sq km**	**Area in percentage (%)**	**Area in sq km**	**Area in percentage (%)**	**Area in sq km**	**Area in percentage (%)**
Agriculture	180160.3	26.89	177307.1	26.46	-2853.18	-1.58
Forest	202289.9	30.19	206530.3	30.83	4240.44	2.1
Grassland	225905.7	33.72	227249.1	33.92	1343.43	0.59
Wetland	623	0.09	648.09	0.1	25.11	4.03
Settlement	441.9	0.07	1724.13	0.26	1282.23	290.16
Shrubland	11230.8	1.68	8012.16	1.2	-3218.67	-28.66
Sparse vegetation	1169.2	0.17	1112.49	0.17	-56.7	-4.85
Bare area	14878.2	2.22	14758.92	2.2	-119.25	-0.8
Water	17110.3	2.55	16466.85	2.46	-643.41	-3.76
Permanent Snow Cover	16186	2.42	16185.96	2.42	0	0
TOTAL	669995.1	100	669995.1	100	**-**	**-**
**(b)**						
**Countries/State**	**2015**	**2016**	**2017**	**2018**	**2019**	**2020**
Bangladesh	25690.49	28450.36	29630.63	27762.42	27762.42	41266
Bhutan	37.83	113.88	51.61	48.29	48.29	30.07
China	1084.18	3633.9	520.58	1092.28	1092.28	468.68
Nepal	13.2	21.42	17.15	7.17	7.17	2.49
Arunachal Pradesh	169.26	418.28	110.11	217.38	217.38	363.63
Assam	4668.07	8674.66	7585.84	10173.78	10172.78	8920.18
Meghalaya	115.56	449.76	178.64	277.67	277.67	189.82
Nagaland	21.64	99.22	38.87	57.7	57.7	35.07
Sikkim	10.33	3.99	6.73	5.39	5.39	2.52
West Bengal	1369.44	2828.53	2482.85	2190.93	2190.93	2446.54

**Fig 2:**
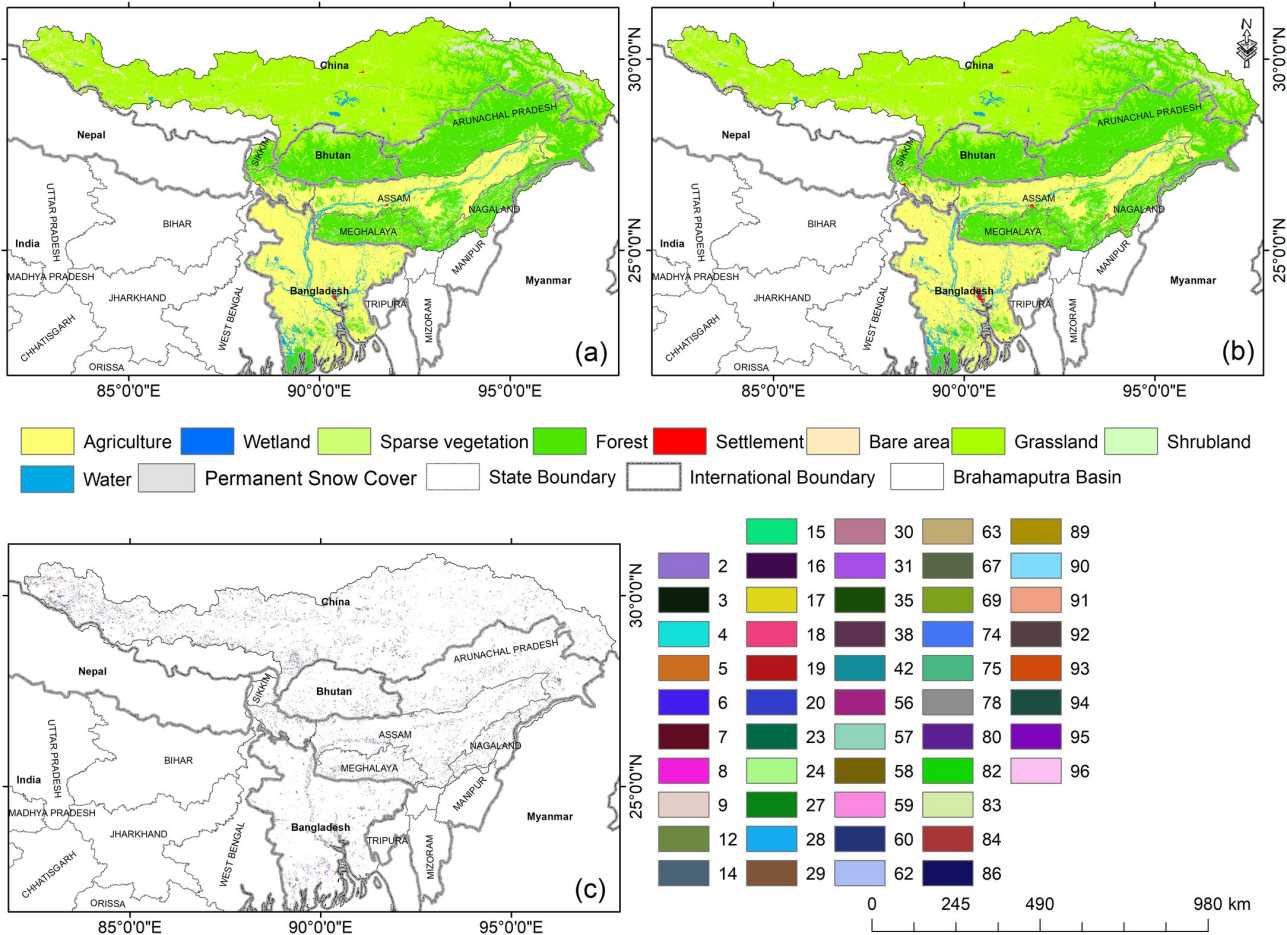
ESA-CCI Land use/land cover map of (a) 2000 (b) 2019 (c) Land Use Transformations (LUT) with change code. Details of the change code discussed in S1 Table.

The major land use transformation was observed from shrubland to forest cover (2786.04 km^2^), followed by agriculture to forest cover (2761.02 km^2^), bare land to grassland (2116.62 km^2^), agricultural land to grassland (1580.76 km^2^), grassland to bare land (1575.9 km^2^), agricultural land to settlement (1123.38 km^2^), forest cover to agricultural land (975.06 km^2^), shrubland to agricultural land (859.5 km^2^), grassland to agriculture (824.67 km^2^) and grassland to forest cover 400.41 km^2^ (Fig 2C, Table 1A). The major increase of built-up land evidently found in major urban centres including Dhaka, Tezpur, Guwahati, Lakhimpur, Dibrugarh, Tinsukia, Shillong, and Itanagar. while the wetland primarily increased in the lower part of Arunachal Pradesh and Assam region altering agricultural land (0.36 km^2^), shrubland (0.09 km^2^), forest (33.3 km^2^), and water body (2.61 km^2^). The increase of forest cover was evident in southern Bangladesh including Dhaka, Barisal, Khulna, Chittagong, parts of Assam (Karbi Anglong, North Cachar Hills, Kamrup-Guwahati, Bongaigaon, and eastern part of Dhubri), Meghalaya (southern part of West Garo Hills, East Garo Hills, South Garo Hills, West Khasi Hills and East Khasi Hills, and small portion in the Jaintia Hills), Nagaland (Wokha, Mokokchung, Mon, Zunheboto, Dimapur, and Kohima), Arunachal Pradesh (Tirap, Changlang, Lohit, Lower Dibang Valley, East-West Siang, Subansiri and small area of Kameng) and southern hills of Bhutan.

### 4.2 Analysing precipitation and river discharge in Brahmaputra basin

**Fig 3:**
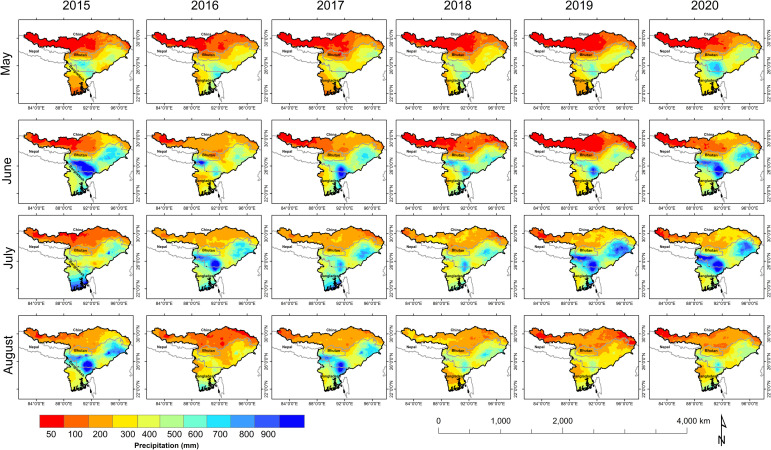
GPM based monthly precipitation in the Brahmaputra Basin during May–August (2015–2020).

The GPM final run-based daily precipitation data were used to deduce cumulative monthly variation of precipitation pattern in the Brahmaputra Basin 2015–2020 (May to August). The study exhibited a spatial variability in cumulative monthly precipitation pattern with extremely high precipitation (>2000 mm) in the lower parts of the basin in contrast to low precipitation (~50–200 mm) in upperparts. Very high precipitation (>900 mm) was observed in highly mountainous terrain area in the lower Brahmaputra Basin (mainly Meghalaya) during June 2015, while the high precipitation (600 to >900 mm) was observed in the major parts of the lower Brahmaputra Basin during June to August 2015, and low precipitation (200 mm– 600 mm) in the part middle part of Brahmaputra Basin and SW Bangladesh and very low precipitation (<200 mm) in the upper Brahmaputra Basin during May to August 2015 (Fig 3). In 2016, there very high precipitation was observed in the Assam valley and some NE Bangladesh during July while a high precipitation in a major part of NE India (Assam valley) and south Bangladesh, alternatively the low (300 – 600mm) precipitation in the entire lower and central part of Brahmaputra Basin (Fig 3). In 2017, high precipitation was observed mainly in the Assam valley and upper parts of Bangladesh during June and August. While moderate precipitation (400 - >800 mm) was observed in parts of the lower Brahmaputra Basin from May to August, in contrast to low precipitation (<400 mm) in the upper part Brahmaputra Basin during the monsoonal period. In 2018, a moderately high (400–800 mm) precipitation was observed in the part of lower Brahmaputra Basin except for lower part of Bangladesh region (Fig 3). In 2019, the high precipitation (>900 mm) was observed in the Assam and Arunachal Pradesh valley) and north-eastern part of Bangladesh during June—July, and a low precipitation in the upper part of Brahmaputra Basin and the south-west Bangladesh (Fig 3). In 2020, very high precipitation was observed in lower Assam and Arunachal Pradesh and northern Bangladesh during June and July with high intensity of precipitation, a moderately high (300–700 mm) precipitation was observed in the lower part of Brahmaputra Basin barring southern Bangladesh, alternatively a low precipitation in the central part of Brahmaputra Basin and southern Bangladesh (Fig 3).

**Fig 4:**
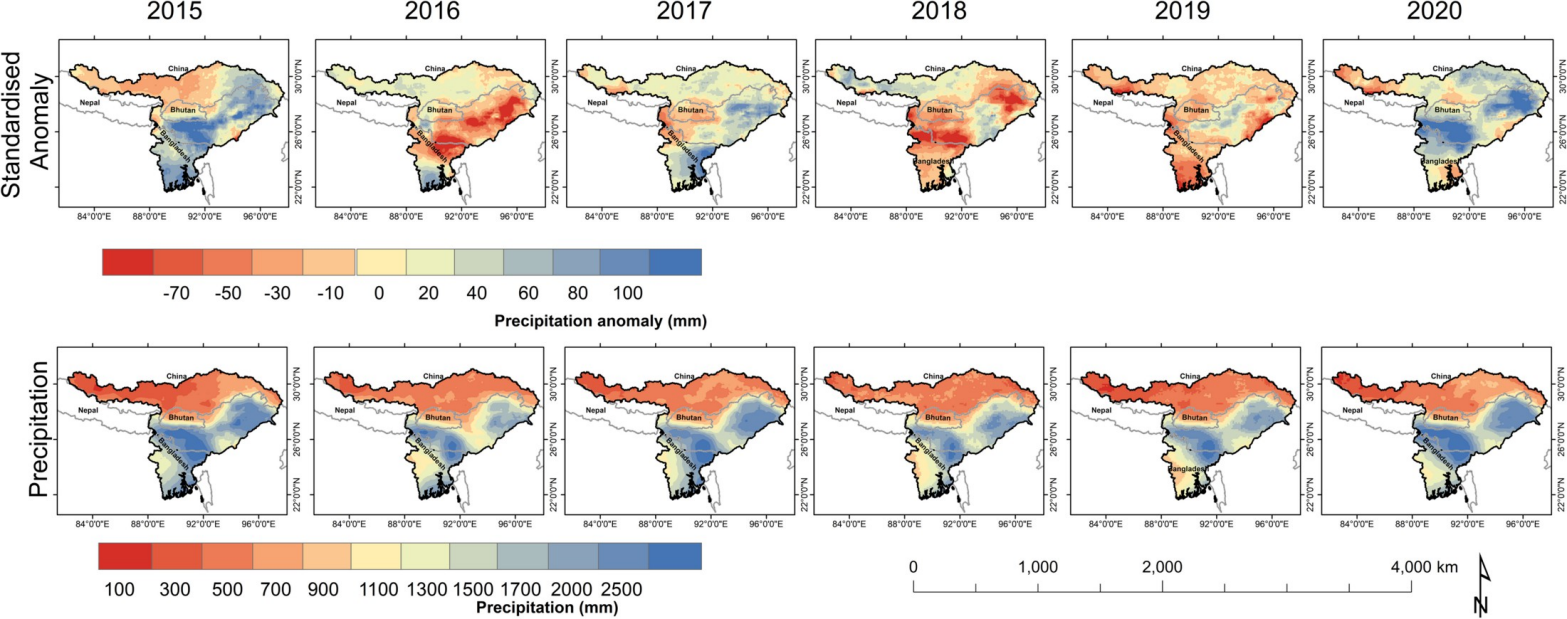
GPM based seasonal precipitation anomaly (upper panel) and seasonal precipitation (lower panel) in the Brahmaputra Basin during May–August (2015–2020).

The precipitation anomaly exhibited a high anomalous precipitation pattern (>2500 mm) in a major part of NE India and NE Bangladesh while a moderate anomalous precipitation pattern (900–2500 mm) in the lower Brahmaputra Basin and the low (100–900 mm) precipitation was observed at the upper part of Brahmaputra Basin from 2015 to 2020 (Fig 4). Highly intensive precipitation was observed in the lower parts of Brahmaputra Basin including Meghalaya, Assam, Arunachal Pradesh, Sylhet, and Dhaka. While the standard anomaly of precipitation during 2015 was observed as the positive anomalous precipitation in the entire lower Brahmaputra Basin with eastern part of Tibet region. In 2016, anomalous precipitation was observed in southern part of Bangladesh while the negative low anomalous precipitation observed in the entire Brahmaputra Basin. In 2017, the primary high anomalous precipitation was observed in the entire Brahmaputra Basin barring the eastern parts of the lower Basin (negative part). In 2018 and 2019, the negative anomalous precipitation (exhibiting reduction in precipitation) was observed in major parts of Brahmaputra Basin barring the upper east and the Assam valley, which recorded positive anomalous precipitation. In 2020, northern Bangladesh, the southern part of NE India, and the eastern part of Tibet had positive precipitation anomalies barring a few regions.

**Fig 5:**
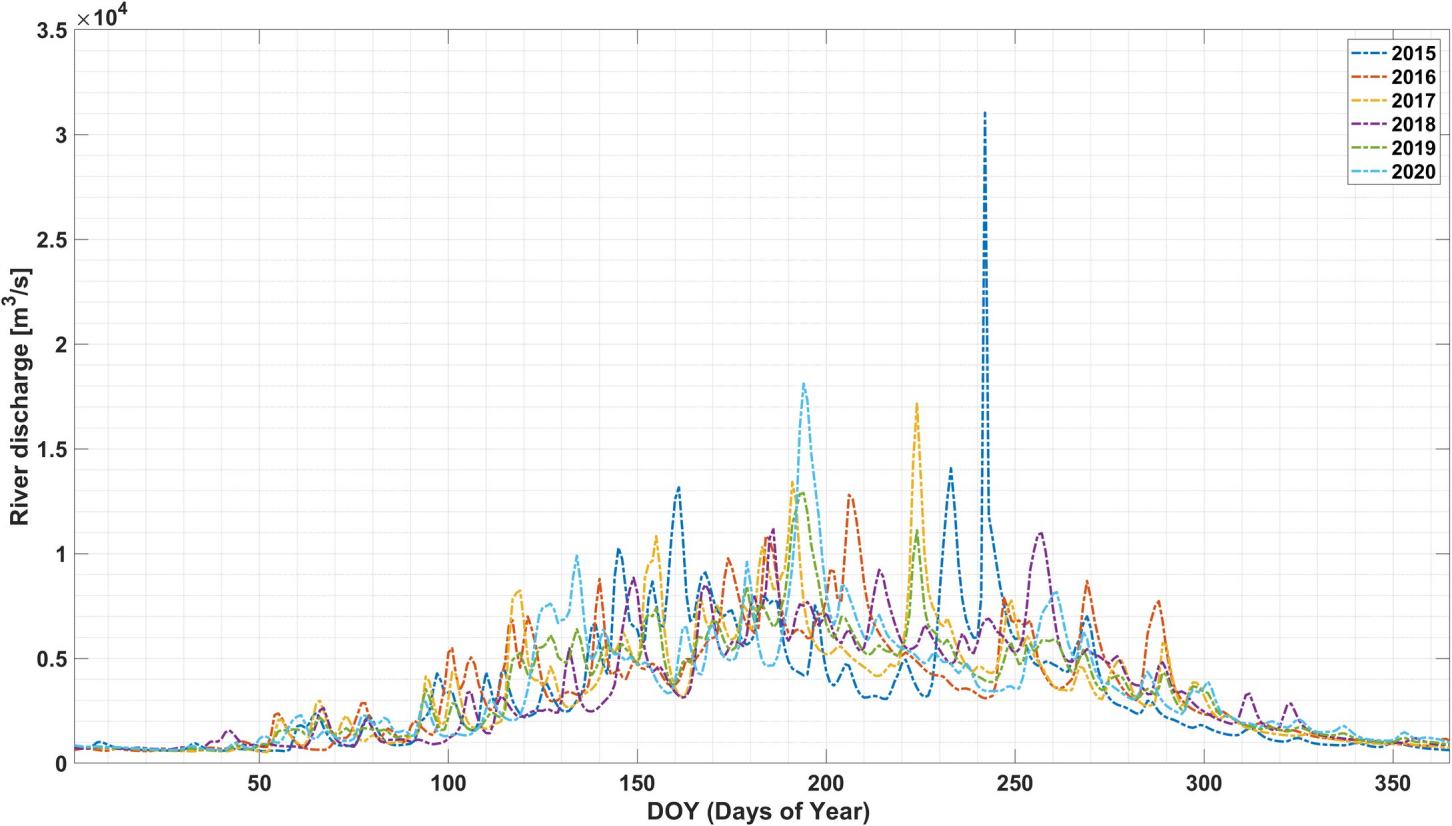
GloFAS ERA-5 based river discharge data of Brahmaputra River (27.4886° N, 95.3558° E).

**Fig 6:**
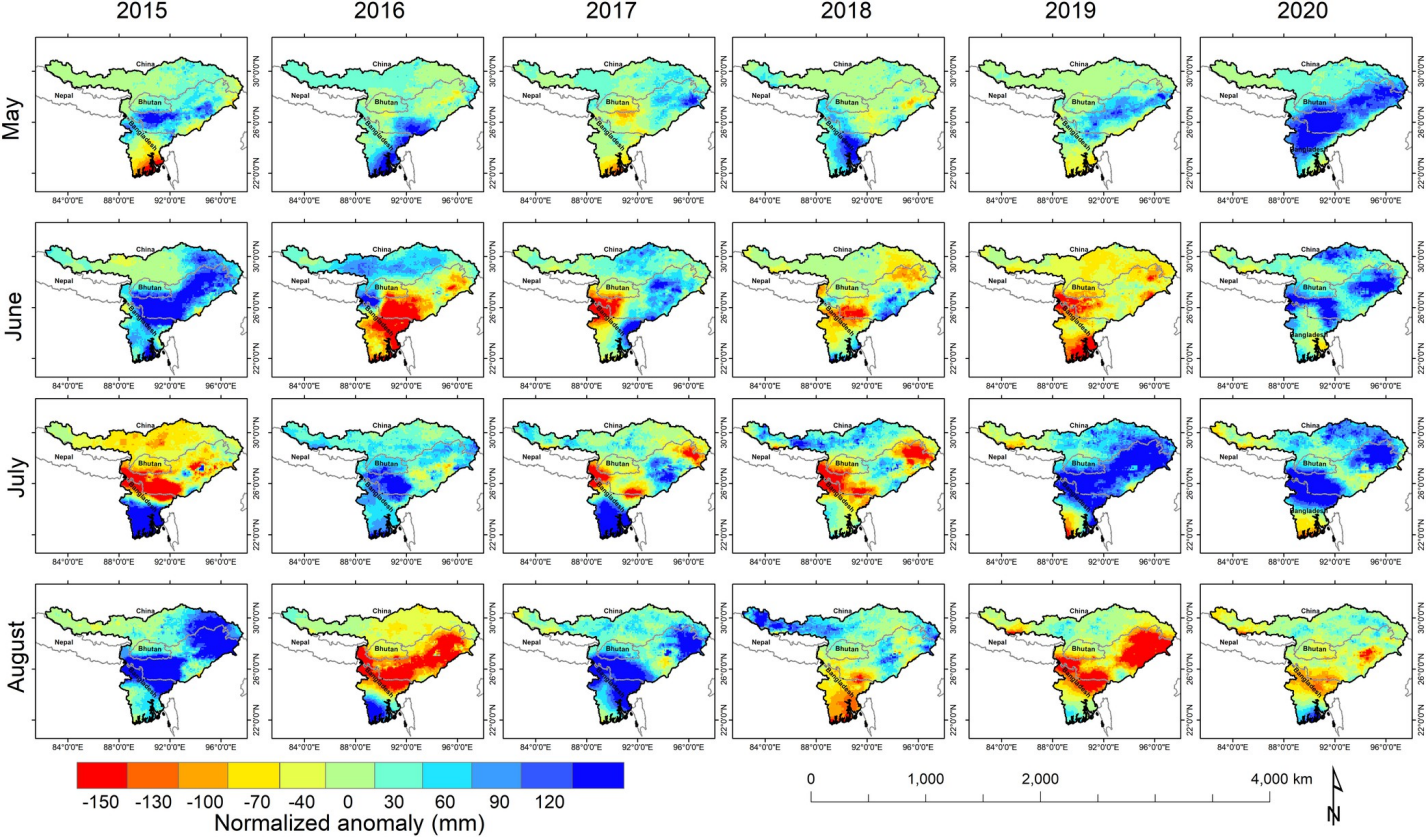
GPM based monthly precipitation anomaly in the Brahmaputra Basin during May–August (2015–2020).

The high river discharge during May to September months are also one of the reasons for flood inundation in the Brahmaputra basin (Fig 5). The high discharge (16,000 to– 18,000 m^3^s^-1^) during August 2017 and July 2020 is analogous to the high precipitation in the region (Fig 6). The study highlights that the river discharge depends on the precipitation concentrations as observed during precipitation events primarily in upper catchment and accumulation in lower catchment ultimately leading to floods in the lower Brahmaputra basin. The positive normalized anomalous precipitation comprised a large area in the months of May, June, and August, but later in July, the negative normalized anomalous precipitation was recorded. High increasing levels mainly showed in the central parts in Brahmaputra Basin, alternatively, in July the middle part highly decreased and the upper part steadily. In 2017, high variations of precipitation were observed in monsoonal periods except for August because it was observed in highly positive normalized precipitation. While in 2019, the positive normalized precipitation was observed in May and July months at the core flood-prone zone of the study area.

### 4.3 Impact of flood inundation in Brahmaputra basin

**Fig 7:**
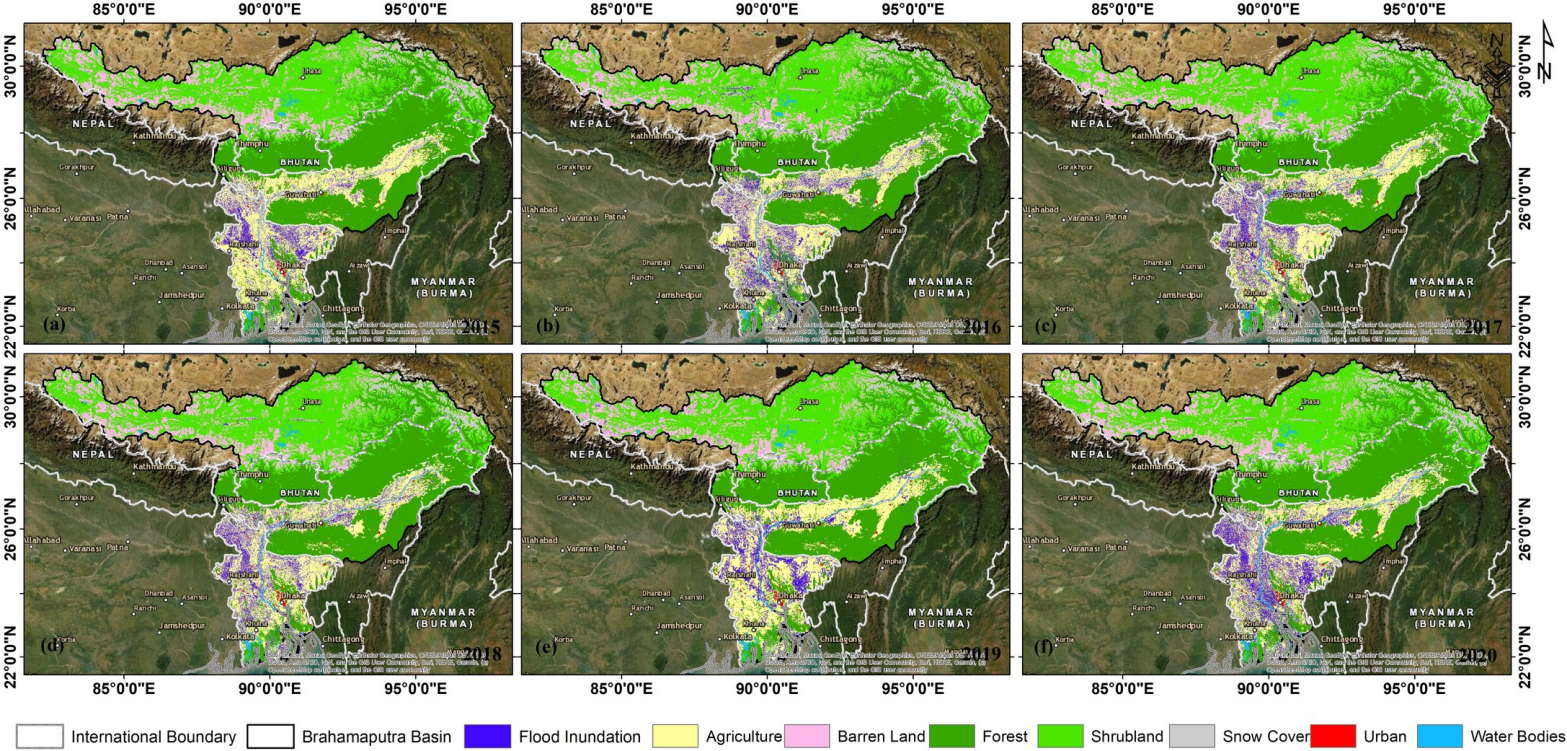
Impact of flood inundation on ESA-CCI LULC for Brahmaputra Basin during (a) 2015 (b) 2016 (c) 2017 (d) 2018 (e) 2019 (f) 2020. Contains information from OpenStreetMap and OpenStreetMap Foundation, which is made available under the Open Database License.

The Brahmaputra River has a long history of flood leading to large destruction almost every year and therefore, the study highlights the use of SAR multi-temporal datasets with precipitation and river discharge to monitor the flood inundation. During late summer and monsoon melting of glaciers and high precipitation especially in the north-eastern part of India accumulate more water in river and leads to very high river discharge, leading to flood inundation in the lower part of basin. The cumulative impact of flood inundation was significantly observed on agricultural land with highest inundation during 2017 (36952.45 km^2^; 90.96% of total LULC in the basin) and minimum during 2016 (34790.22 km^2^; **77**.84%). Forest is the second most affected flood with a highest inundation during 2016 (5.40%; 2413.12 km^2^) and a lowest inundation during 2017 (2.74%; 1111.72 km^2^) disturbed the biodiversity, farming, and also wildlife (Fig 7). In contrast, the settlement areas were slightly low in coverage but had large impacts on mankind and were primarily affected during 2016 (1758.19 km^2^), and least during 2018 (426.42 km^2^) (Table 2; Fig 7). Although the large parts of the Brahmaputra basin are under cultivation and have been the largest impacts due to flood inundation. Bangladesh is mainly covered by rivers and the majority of its parts are affected by the flood inundation during monsoon and post-monsoon periods. In 2020, Bangladesh’s highly affected area is 41266 km^2^ and the lowest affected area is in 2015, 25690.49 km^2^. And then in India Assam valley was invaded by floods generally each year 2018 and 2019 were the most impactful times for Assam (10173.78 km^2^ and 10172.78 km^2^), but in 2020 it was the least affected 8920.18 km^2^. West Bengal was also oppressed by floods in most of the year, in 2016 there was a high impact of (2828.53 km^2^) and then it slowly lessened towards 2020. In contrast, flood inundation has a lower impact on the area of China, Bhutan, and some parts of NE India (Meghalaya, Arunachal Pradesh) countries due to its safer site and situation (Table 1B).

**Table 2:** Impact of flood inundation on different land-use/land-cover types in Brahmaputra Basin using Sentinel 1A/1B SAR.

Years	2015		2016		2017		2018		2019		2020	
Features	Area (km^2^)	Area	Area (km^2^)	Area	Area (km^2^)	Area	Area (km^2^)	Area	Area (km^2^)	Area	Area (km^2^)	Area
		(%)		(%)		(%)		(%)		(%)		(%)
Agriculture	28406.81	85.61	34790.22	77.84	36952.45	90.96	36871.59	88.14	36892.35	88.19	48182.59	89.68
**Forest**	1570.29	4.73	2413.12	5.4	1111.72	2.74	1409.04	3.37	1407.85	3.37	2257.66	4.2
**Settlement**	911.8	2.75	1758.19	3.93	426.42	1.05	411.85	0.98	412.12	0.99	612.25	1.14
**Shrubland**	1342.04	4.04	3615.14	8.09	1264.72	3.11	1428.13	3.41	1461.69	3.49	1581.28	2.94
**Bare Land**	736.07	2.22	1858.07	4.16	670.95	1.65	1451.79	3.47	1319.05	3.15	787.67	1.47
**Water Bodies**	213.3	0.64	258.83	0.58	197.14	0.49	260.11	0.62	339.34	0.81	303.46	0.56
**Total**	**33180.29**	**100**	**44693.58**	**100**	**40623.4**	**100**	**41832.52**	**100**	**41832.41**	**100**	**53724.9**	**100**

## 5. Conclusion

Flood is one of the most common phenomena in the Brahmaputra Basin affecting a large area, assets, and population during monsoon and post-monsoon periods. The present study highlights the spatio-temporal patterns of flood inundation in the entire Brahmaputra Basin analysing its main causative factors *viz*., cumulative precipitation patterns (2015 to 2020). The study exhibited a high (900–2000 mm) to very high (>2000 mm) monthly cumulative precipitation in the part of NE India (Mawsynram of Khasi hill in Meghalaya) and NE Bangladesh in the Brahmaputra Basin. The normalized precipitation indicated a rise in precipitation patterns in the Central and lower parts of the Brahmaputra Basin during 2015–2020. The temporal optical satellite data based LULC change exhibited a significant decline in shrubland (28.66%, 3218.67 km^2^), in contrast to a significant rise in settlement (290.16%; 1282.23 km^2^) in the region. The high to the moderate impact of flood inundation observed in Bangladesh (vary between 63% to 78% compared to total inundation in the basin); Assam (vary between 14% to 25% compared to total inundation in the basin), and West Bengal (vary between 4% to 6.5% compared to total inundation in the basin). The flood had severely affected ~85% of total agricultural land, and ~2% of settlements in the Brahmaputra Basin during the observation periods. Although the lower Brahmaputra Basin has a long history of flood inundation, the recent built-up growth and significant land use transformation exacerbate flood vulnerability and risk to a greater extent. Thus, there is an urgent need to reduce the increasing impact of floods by adopting proper afforestation measures in the upper catchments to reduce the soil erosion; the de-siltation process of the Brahmaputra River in Assam and Bangladesh to increase the depth of the river. Nevertheless, the region is prone to flood, and thus the controlled built-up development is pertinent. Settlements in the study region are surrounded by enumerable operational holdings of varying shapes and sizes. This risk shows high population pressure on arable land that leads to further fragmentation. The study will help to contribute towards sustainable land use planning and management towards the protection of extremely rich biodiversity of the Brahmaputra valley.

## Supporting information

S1 Table Area (in km^2^) transformation and code for LUT of LULC corresponding to year 2001 from 2019 as shown in Fig 2. (DOCX)
