# Supplementary material for: Analysing frequent extreme flood incidences in Brahmaputra basin, South Asia
Source: PLoS One. 2022 Aug 22;17(8):e0273384. doi: 10.1371/journal.pone.0273384 (PMC9394833; doi:10.1371/journal.pone.0273384)
Supplement: S1 Table — (DOCX) [file pone.0273384.s001.docx]

Table S1: Area (in km^2^) transformation and code for LUT of LULC corresponding to year 2001 from 2019 as shown in Figure 2.

| **Class** | **Reference Class** | **New Class** | **Area Change** | **Land Use Land Cover Transformation** | **Class** | **Reference Class** | **New Class** | **Area Change** |
| --- | --- | --- | --- | --- | --- | --- | --- | --- |
| 2 | Agriculture | Forest | 2761.02 |  | 56 | Shrubland | Agriculture | 859.5 |
| 3 | Agriculture | Grassland | 1580.76 |  | 57 | Shrubland | Forest | 2786.04 |
| 4 | Agriculture | Wetland | 0.36 |  | 58 | Shrubland | Grassland | 12.42 |
| 5 | Agriculture | Settlement | 1123.38 |  | 59 | Shrubland | Wetland | 0.09 |
| 6 | Agriculture | Shrubland | 12.51 |  | 60 | Shrubland | Settlement | 4.41 |
| 7 | Agriculture | Sparse vegetation | 11.25 |  | 62 | Shrubland | Sparse vegetation | 1.71 |
| 8 | Agriculture | Bare area | 165.06 |  | 63 | Shrubland | Bare area | 2.34 |
| 9 | Agriculture | Water | 200.88 |  | 67 | Sparse vegetation | Agriculture | 3.15 |
| 12 | Forest | Agriculture | 975.06 |  | 69 | Sparse vegetation | Grassland | 319.23 |
| 14 | Forest | Grassland | 313.56 |  | 74 | Sparse vegetation | Bare area | 42.12 |
| 15 | Forest | Wetland | 33.3 |  | 75 | Sparse vegetation | Water | 0.27 |
| 16 | Forest | Settlement | 31.23 |  | 78 | Bare area | Agriculture | 21.96 |
| 17 | Forest | Shrubland | 431.1 |  | 80 | Bare area | Grassland | 2116.62 |
| 18 | Forest | Sparse vegetation | 1.71 |  | 82 | Bare area | Settlement | 1.8 |
| 19 | Forest | Bare area | 0.18 |  | 83 | Bare area | Shrubland | 0.72 |
| 20 | Forest | Water | 24.57 |  | 84 | Bare area | Sparse vegetation | 110.43 |
| 23 | Grassland | Agriculture | 824.67 |  | 86 | Bare area | Water | 20.7 |
| 24 | Grassland | Forest | 400.41 |  | 89 | Water | Agriculture | 317.7 |
| 27 | Grassland | Settlement | 87.93 |  | 90 | Water | Forest | 92.79 |
| 28 | Grassland | Shrubland | 2.52 |  | 91 | Water | Grassland | 250.38 |
| 29 | Grassland | Sparse vegetation | 165.78 |  | 92 | Water | Wetland | 2.61 |
| 30 | Grassland | Bare area | 1575.9 |  | 93 | Water | Settlement | 33.21 |
| 31 | Grassland | Water | 192.33 |  | 94 | Water | Shrubland | 0.99 |
| 35 | Wetland | Forest | 10.89 |  | 95 | Water | Sparse vegetation | 17.19 |
| 38 | Wetland | Settlement | 0.27 |  | 96 | Water | Bare area | 367.38 |
| 42 | Wetland | Water | 0.09 |  |  |  |  |  |
